# Dramatic Structural Changes Resulting from the Loss of a Crucial Hydrogen Bond in the Hinge Region Involved in C-Terminal Helix Swapping in SurE: A Survival Protein from *Salmonella typhimurium*

**DOI:** 10.1371/journal.pone.0055978

**Published:** 2013-02-07

**Authors:** Yamuna Kalyani Mathiharan, Anju Pappachan, H. S. Savithri, Mathur R. N. Murthy

**Affiliations:** 1 Molecular Biophysics Unit, Indian Institute of Science, Bangalore, India; 2 Department of Biochemistry, Indian Institute of Science, Bangalore, India; University of Canterbury, New Zealand

## Abstract

Domain swapping is an interesting feature of some oligomeric proteins in which each protomer of the oligomer provides an identical surface for exclusive interaction with a segment or domain belonging to another protomer. Here we report results of mutagenesis experiments on the structure of C-terminal helix swapped dimer of a stationary phase survival protein from *Salmonella typhimurium* (*St*SurE). Wild type *St*SurE is a dimer in which a large helical segment at the C-terminus and a tetramerization loop comprising two β strands are swapped between the protomers. Key residues in *St*SurE that might promote C-terminal helix swapping were identified by sequence and structural comparisons. Three mutants in which the helix swapping is likely to be avoided were constructed and expressed in *E. coli*. Three-dimensional X-ray crystal structures of the mutants H234A and D230A/H234A could be determined at 2.1 Å and 2.35 Å resolutions, respectively. Contrary to expectations, helix swapping was mostly retained in both the mutants. The loss of the crucial D230 OD2– H234 NE2 hydrogen bond (2.89 Å in the wild type structure) in the hinge region was compensated by new inter and intra-chain interactions. However, the two fold molecular symmetry was lost and there were large conformational changes throughout the polypeptide. In spite of these changes, the dimeric structure and an approximate tetrameric organization were retained, probably due to the interactions involving the tetramerization loop. Mutants were mostly functionally inactive, highlighting the importance of precise inter-subunit interactions for the symmetry and function of *St*SurE.

## Introduction

Protomers of protein oligomers are almost always related by nearly exact point group symmetries. Formation of oligomeric structures allow proteins of large size to be coded by relatively small genes which are less prone to lethal mutations compared to larger genes. Oligomeric structure is also crucial for key functions such as allostery [1] and half of the sites reactivity [2]. The mechanism by which proteins acquire symmetric oligomeric forms needs to be experimentally and theoretically investigated. It has been proposed that symmetrical oligomeric forms evolve through intermediate states stabilized by domain swapping interactions [3], [4]. In domain swapping, each protomer provides an interacting surface for exclusive interaction with another protomer. The term domain swapping was coined by David Eisenberg [5] for describing the structure of diphtheria toxin dimer. In this dimer, C-terminal receptor binding domains are exchanged between the two interacting protomers. Apart from the unique way of oligomerization, it is proposed that domain swapping provides a mechanism by which stable unswapped dimers and higher order oligomers of proteins might evolve [4]. Further, studies on domain swapping are important as oligomerization of amyloid proteins that lead to severe neurodegenerative disorders may be a result of similar intermolecular interactions [6]. In particular, it is important to probe the role of domain swapping in protein symmetry, oligomerization and function. In this manuscript, we report such studies on a stationary phase survival protein (SurE) from *Salmonella typhimurium* (*St*SurE).

SurE is one of the several proteins that gets expressed when bacterial cells are subjected to environmental stresses and in the stationary growth phase of bacteria [7]. SurE has been shown to possess phosphatase activity and appears to be specific to nucleoside monophosphates [8], [9]. Sequence alignment of SurE from various prokaryotic sources shows that it is a well conserved protein. The X-ray crystal structures of SurE from the mesophilic organism *Salmonella typhimurium* (*St*SurE [10], PDB ID 2V4N) and thermophilic organisms *Thermotoga maritima* (*Tm*SurE [11], [12], PDB ID 1J9J**)**, *Thermus thermophilus* (*Tt*SurE [9], PDB ID 2E6E), *Aquifex aeolicus* (*Aa*SurE [13], PDB ID 2WQK) and *Pyrobaculum aerophilum* (*Pa*SurE [8], PDB ID 1L5X) have been reported. Recently the structure *Coxiella burnetii* SurE (PDB ID 3TY2) has been deposited in the PDB.

**Figure 1:**
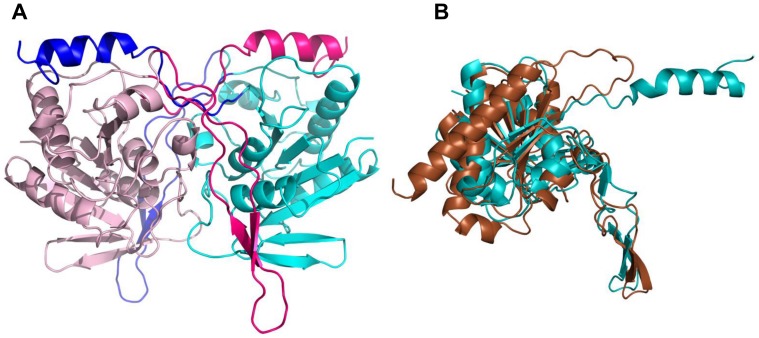
SurE structure. A) Structure of wild type *St*SurE dimer shown in ribbon representation. Chain A is in light pink while chain B is in light blue. The swapped C-terminal helices (residues 227–253) and tetramerization loops (residues 179–210) of A and B chains are shown in darker shades. B) Structural superposition of *St*SurE (blue) and *Pa*SurE (brown) protomers. C-terminal helices are swapped in *St*SurE but not in *Pa*SurE, where a reversal of polypeptide that occurs before the C-terminal helix could be observed.

*St*SurE polypeptide consists of 253 amino acid residues and including the N-terminal segment containing the hexahistidine tag from pRSETC vector is of MW 28.5 kDa. The structure of *St*SurE has been determined at 1.7 Å resolution [10]. The *St*SurE protomer has a Rossmann fold like structure similar to those of its thermophilic homologs (Fig. 1a). It is a αβα sandwich consisting of 13 β-strands, six α-helices and three 3_10_ helices. The N-terminal half (residues 1–125) of SurE proteins is more conserved when compared to the C-terminal half (residues 126–253). The residues important for the phosphatase function are in the N-terminal half. *St*SurE exists in a dimeric form in solution. The crystal structure of *St*SurE reveals that the C-terminal helices of the two protomers of the dimer are swapped. The interactions of the C-terminal helix of one protomer are exclusively with atoms of the other protomer. Interestingly, out of the six SurE X-ray structures that have been determined, five exhibit C-terminal helix swapping. Only in *Pa*SurE, this swapping is mostly avoided and the C-terminal helix reverts back to the same protomer making interactions that are similar to those of domain swapped dimers (Fig. 1b [8]). Apart from the C-terminal helix, a tetramerization loop consisting of a pair of β-strands is also swapped between the two protomers (Fig. 1a) in all the six SurE structures. In most SurEs, two C-terminal helix swapped dimers are held together forming a loose tetramer by interactions involving residues of the tetramerization loop. Therefore, the tetrameric forms observed in the crystal structures may have physiological significance.

*St*SurE was selected for studies on domain swapping as there is at least one homologous structure in which swapping of the C-terminal helices appears to have been avoided without leading to the loss of oligomeric structure or function. It was of interest to examine if an unswapped dimer of *St*SurE resembling *Pa*SurE dimer could be constructed by mutagenesis. D230 and H234 of *St*SurE were identified as important residues for helix swapping by structure based sequence comparison of SurE homologs. Structural studies on mutants of these residues revealed large conformational changes throughout the polypeptide and loss of exact two fold symmetry between the protomers, although dimeric organization and domain swapping were retained. The resulting dimers were mostly functionally inactive. These dramatic structural changes that result from replacement of key residues involved in domain swapping suggest that although the folded structures of proteins tolerate mutations to the point of losing identifiable sequence similarities with homologs, certain key residues cannot be replaced without disruption of the structure, symmetry and function. The results represent systematic experimental investigations on the importance of precise domain swapping interactions for the structure and function of *St*SurE.

## Results

### Identification of Residues Promoting C-terminal Helix Swapping in *St*SurE

The residues that are likely to be important for C-terminal helix swapping in *St*SurE were identified by examining the structures and sequences of SurEs from different organisms (Supplementary Fig. S1). C-terminal helix swapping does not occur in *Pa*SurE. The conformation of the segment 242–245 of *Pa*SurE is very different from that of the corresponding residues of *St*SurE (Fig. 1b) and other domain swapped SurEs. A polypeptide turn in this segment that occurs only in *Pa*SurE brings the C-terminal helix back to the same protomer while the corresponding helix in other SurEs extends to the two fold related subunit. The interactions of the C-terminal helix of *Pa*SurE with the protomer to which it belongs are very similar to the interactions in other SurEs between the corresponding helix and the symmetry related protomer. This difference is most likely a result of amino acid replacements in the segment where *Pa*SurE has a chain reversal (Fig. 1b). Examination of the sequences of this segment in *St*SurE and *Pa*SurE (Supplementary Fig. S1a) suggested that D230 and H234 of *St*SurE might be the key residues responsible for C-terminal helix swapping. D230 is involved in several interactions in *St*SurE [10]: OD1of D230 is hydrogen bonded to N of T232 (2.95 Å), OD1 of D230 is hydrogen bonded to OG1 of T232 (2.66 Å) and OD2 of D230 is hydrogen bonded to NE2 of H234 (2.89 Å) (Supplementary Fig. S1b). D234 of *Aa*SurE [13] is structurally equivalent to D230 of *St*SurE and is involved in similar interactions (Supplementary Fig. S1c). The hydrogen bond between D234 and Y238 (2.54 Å) of *Aa*SurE is analogous to the hydrogen bond between OD2 of D230 and NE2 of H234 (2.89 Å) of *St*SurE (Supplementary Figures S1b & c). These hydrogen bonds appear to prevent the sharp turn needed in this segment for the formation of an unswapped dimer. Therefore mutation of D230 or H234 or both in *St*SurE may lead to a structure in which C-terminal helix swapping is avoided. Towards this goal, mutants H234A, D230A and D230A/H234A were constructed and expressed in *E. coli*.

### Characterization of the Mutants

**Figure 2:**
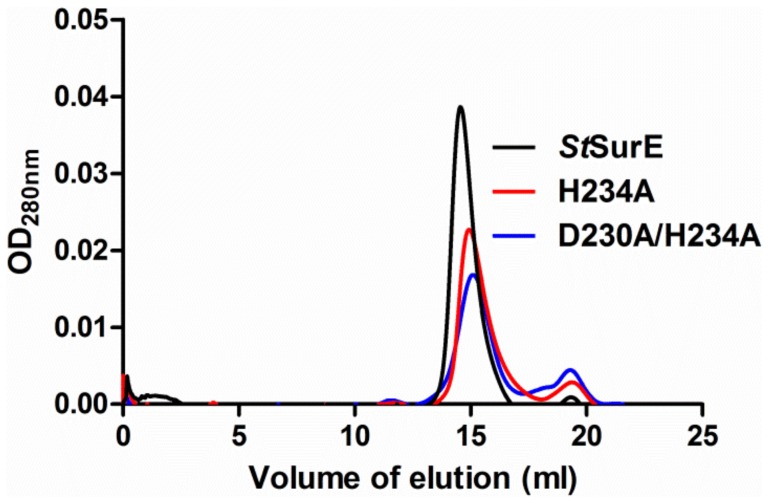
Characterization of the *St*SurE mutants. Gel filtration profile showing wild type, H234A and D230A/H234A mutant proteins eluting around 15 ml, which corresponds to molecular weight of a dimer (57 kDa).

**Figure 3:**
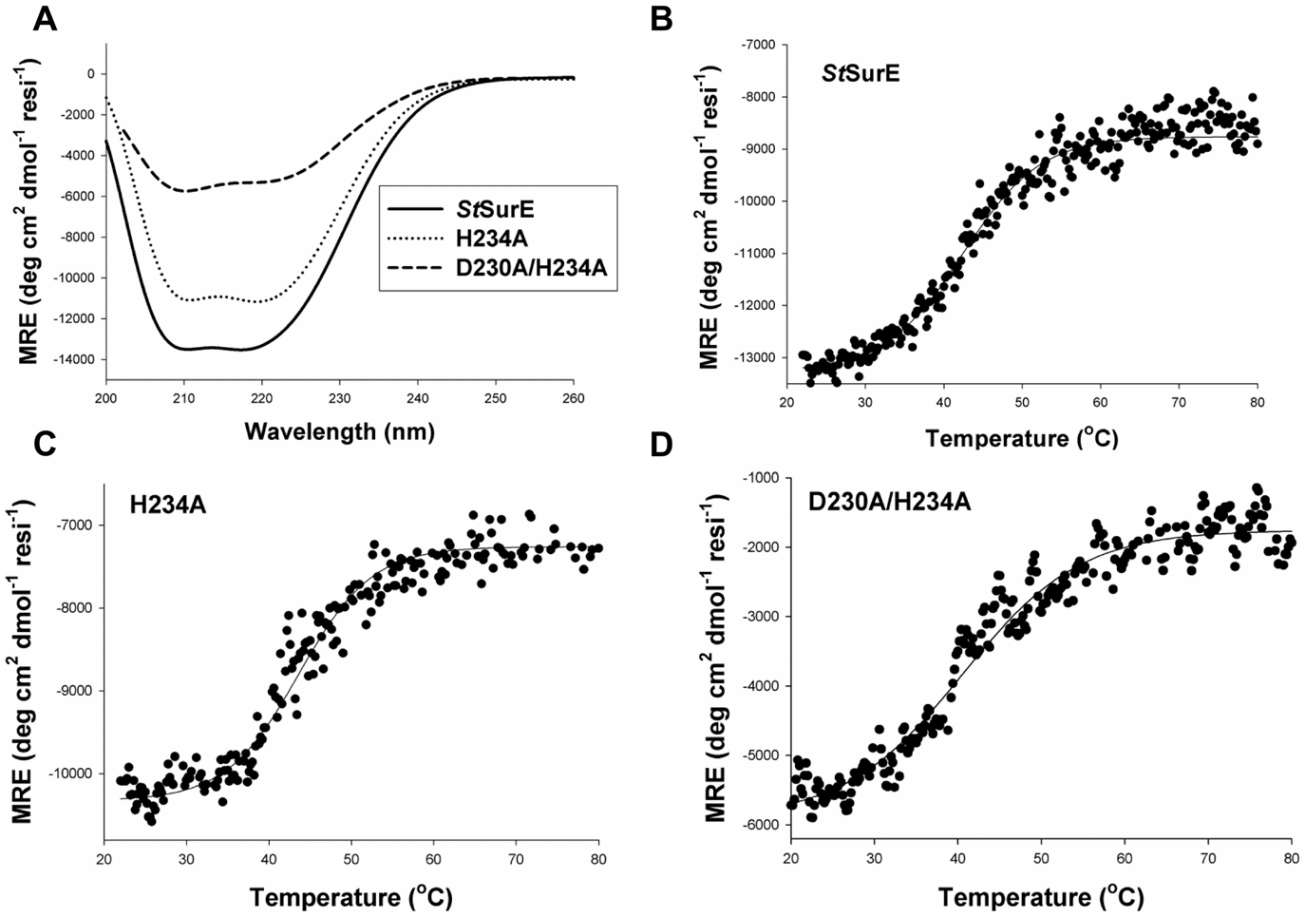
Circular Dichorism (CD) studies of *St*SurE mutants. A) CD spectra exhibited minima at 208 and 222 nm indicating that the native and the mutants are α/β proteins. B), C) and D) show melting profiles monitored by CD at 208 nm as a function of temperature for native (T_m_ = 43°C), H234A (T_m_ = 43°C) and D230A/H234A (T_m_ = 41°C), respectively in HEPES buffer. Note the broad melting profile of the double mutant.

H234A, D230A and D230A/H234A mutants could be expressed in soluble forms and purified to homogeneity following a protocol similar to that used for the wild type enzyme. The yield of D230A was 2–5 fold less than those of other mutants and the purified protein had a tendency to fall out of solution. Hence this mutant was not used for further studies. SurE homologs are known to exist either as dimers or tetramers in solution [8], [9], [11], [12], [13]. Gel filtration studies on mutant *St*SurEs revealed that they are dimeric in solution (Fig. 2). Dynamic light scattering (DLS) studies for H234A were also consistent with a dimeric form (data not shown). CD spectra of H234A and D230A/H234A mutants exhibited minima at 208 nm and 222 nm, as reported for the wild type enzyme (Fig. 3a). The stabilities of the mutants were measured by recording CD at 208 nm as a function of increasing temperature. The T_m_ of native SurE, H234A and D230A/H234A mutants in HEPES buffer (pH 7.5) were 43°C, 43°C and 41°C, respectively (Fig. 3b, c & d). Thus, the mutations have not resulted in the destabilization of the polypeptide fold or dimeric structure. Comparison of the thermal melting profiles of the wild type and mutant SurEs (Fig. 3) suggests that the double mutant with a broad melting profile might represent a larger ensemble of structures in solution.

*St*SurE has been shown to possess alkaline phosphatase activity with the substrate pNPP with a pH optimum of 7 [10]. Alkaline phosphatase activities of the native and mutant enzymes were estimated following the procedure described in materials and methods. The activity of H234A was only 5% of that of the native enzyme. The double mutant D230A/H234A had no detectable activity (<0.1%).

### Crystallization of Mutant Proteins

H234A and the double mutant D230A/H234A could be crystallized under conditions described in materials and methods. These crystals belonged to the orthorhombic space group C222_1_. The structure of wild type *St*SurE has been determined in two different forms belonging to monoclinic C2 and orthorhombic F222 space groups, respectively [10]. It was found that the native enzyme could also be crystallized under the new condition found suitable for obtaining crystals of mutant enzymes. However, unlike the mutant crystals, these crystals belonged to the orthorhombic space group F222 and were isomorphous with the crystals obtained by Pappachan *et al.* (2008). The unit cell parameters of the two mutants were nearly identical and the volume of their asymmetric units was compatible with two protomers (Table 1). The calculated Matthews coefficients for H234A *St*SurE and D230A/H234A *St*SurE were 2.22 A^3^ Da^−1^ and 2.19 A^3^ Da^−1^, respectively. In contrast, the monoclinic and orthorhombic forms of native *St*SurE contained 4 and 1 protomers, respectively, in the crystal asymmetric unit.

**Table 1:** Data Collection and Refinement Statistics.

	H234A	D230A/H234A
**a, b, c (Å)**	81.04, 98.14, 127.54	80.60, 97.33, 127.15
**α, β, γ (°)**	90,90,90	90,90,90
**Space group**	C222_1_	C222_1_
**Resolution (Å)**	40.52 −2.12 (2.23 −2.12)	55.78 − 2.35 (2.48 − 2.35)
**Total No. of reflections**	1,87,111 (26,998)	1,10,137 (15,984)
**No. of unique reflections**	29,206 (4,191)	21,227 (3,043)
**Multiplicity**	6.4 (6.4)	5.2 (5.3)
**Mean (I/σ(I))**	19.1 (4)	14.6 (3.3)
**R _merge_** a	5.7 (49.9)	7.3 (48.2)
**Completeness (%)**	100 (100)	100(100)
**Protomers in ASU**	2	2
**Solvent content (%)**	44.7	43.8
**PDB Code**	4G9O	4GAD
**Refinement Statistics**		
**R_Work_** b **(%)**	21.94	22.03
**R_Free_** b **(%)**	27.29	26.89
**No. of Atoms**		
**Protein**	A- 1795	A- 1752
	B- 1813	B- 1730
**Water**	248	230
**Ethylene glycol**	28	–
**Glycerol**	–	12
**Mg^2+^**	2	2
**RMSD from Ideal Values**		
**Bond angle (°)**	1.003	1.137
**Bond length (Å)**	0.005	0.006
**Residues in Ramachandran Plot (%)**		
**Most allowed region**	90.3	88.3
**Allowed region**	9.7	11.7

a R_merge_ = (Σ_hkl_Σ_i_ |I_i(hkl)_-<I_hkl_>|)%Σ_hkl_Σ_i_I_i(hkl)_, where I_i(hkl)_ is the intensity of the i^th^ measurement of reflection (hkl) and <I_hkl_> is its mean intensity.

b R_work_ = (Σ_hkl_||Fobs|-|Fcalc||)/Σ_hkl_|Fobs|, R_free_ was calculated similarly by using 5% of the reflection that were excluded from refinement.

### Strategy for Structure Determination

The structure of H234A *St*SurE could not be determined by molecular replacement with acceptable scores using the Phaser [14] program and *St*SurE monomer or dimer as phasing models. Even after removing “C-terminal helix” (residues 227–253 [10]) involved in domain swapping, a solution could not be obtained. This suggested that the mutation might have resulted in additional structural changes. Examination of *St*SurE structure suggested that the swapped “tetramerization loop” (residues 179–210 [10]) and the amino terminal segments are likely to be flexible and could have altered conformations in the mutants. Therefore, residues corresponding to the tetramerization loop (residues 179–210) and five N-terminal residues were deleted in addition to residues 227–253. With this truncated model, a solution with an LLG score of 905 was obtained when two copies of the truncated protomer were placed in the asymmetric unit of the H234A *St*SurE. Five rounds of rigid body refinement using REFMAC [15] program of CCP4 suite [16] resulted in R and R-free values of 42.8% and 43.6%, respectively. The structure was further refined with alternative cycles of manual model building using COOT [17], restrained and TLS refinement using REFMAC [15]. Particular care was taken in modelling the segment involved in domain swapping. Refinement of models with and without swapping resulted in similar refinement statistics. It was however concluded that the helices are mostly swapped based on the following observations.

**Figure 4:**
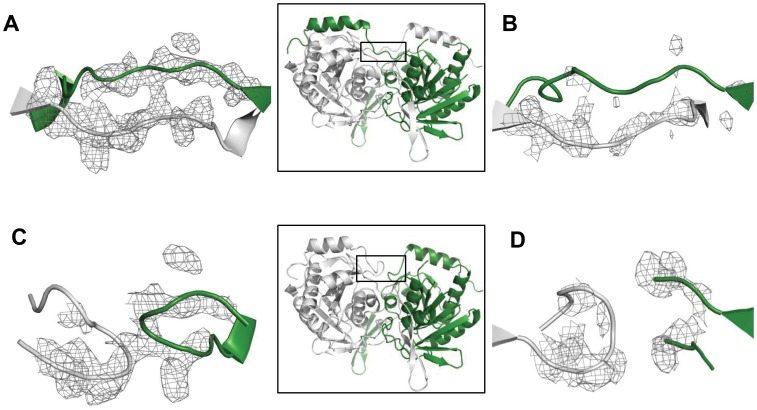
Fit of swapped and unswapped conformations of the hinge region to the electron density map. A) and B) show fit of swapped conformations to the electron density map (Fo-Fc; calculated after omitting the hinge region 227–231) in H234A (contoured at 3σ) and D230A/H234A (contoured at 2.4σ) respectively. C) and D) show fit of segments built as unswapped in H234A (contoured at 3σ) and D230A/H234A (contoured at 2.4σ), respectively. Color code: A chain – gray, B chain – green. In the inserts, hinge regions are boxed.

- The fit of swapped forms to the electron density was better than that of unswapped forms (Fig. 4a and 4c).
- Refinement of the 227–235 segment built in the unswapped conformation left significant unaccounted positive difference density, a few short contacts and some residues in the disallowed region of the Ramachandran map. The same segment built in the swapped form fitted the density better with no short contacts or residues in the disallowed region of the Ramachandran map.

The final model of H234A *St*SurE consists of a dimer in the asymmetric unit with 244 residues (out of 253+14 N-terminal tag) in chain A, 252 residues in chain B, seven molecules of ethylene glycol (added as cryo-protectant), 248 water molecules and two magnesium ions. The R and R-free for the final model are 21.94% and 27.29%, respectively. Examination of the structure by PROCHECK [18] reveals that 90.3%, 9.7% of the residues are in the most allowed and allowed regions of the Ramachandran map.

The structure of D230A/H234A was determined by Molecular Replacement (MR) with Phaser [14] using H234A mutant dimer from which residues corresponding to the C-terminal helix (residues 227–253) were deleted as the phasing model. A solution with an LLG score of 2380 was obtained. Further refinement and model building were carried out with REFMAC [15] and COOT [17], respectively. In this mutant also, the hinge region could be built in both swapped and unswapped forms. Examination of electron density map and geometry of the model revealed that as in H234A, in D230A/H234A mutant also, the C-terminal helices are largely swapped (Fig. 4b & 4d). In this conformation, all residues were in the allowed region of the Ramachandran map.

The final model for D230A/H234A consists of 245 residues in chain A, 244 residues in chain B, 230 water molecules, two glycerol (cryo-protectant) molecules and two magnesium ions. The final R and R-free for the refined model were 22.03% and 26.89% respectively. The refinement statistics for both the structures are given in Table 1. The electron densities for the mutated residues are shown in Supplementary Fig. S2. The coordinates and structure factors of both the mutants have been deposited in the Protein Data Bank (H234A - 4G9O; D230A/H234A - 4GAD).

### Structures of *St*SurE Mutants

The crystallographic asymmetric units of H234A and D230A/H234A *St*SurEs contain two protomers (labelled A and B). The secondary structural elements in these protomers are broadly similar to those of the native protein. Significant differences are observed between the polypeptide structures of the two protomers present in the crystallographic asymmetric units of H234A and D230A/H234A. These differences were analysed by calculating the deviation of the equivalent Cα atoms (Supplementary Fig. S3) upon superposition of the protomer structures using the Superpose program of CCP4 suite [16]. The segments with large differences of more than 5 Å correspond to the swapped helical segment (residues 227–253) and the tetramerization loop (residues 179–210). In the wild type monoclinic *St*SurE structure also, these segments show larger differences among the four subunits of the crystal asymmetric unit. However, the deviations are small and the largest deviation observed for the tetramerization loop is less than 3 Å (Supplementary Fig. S3a). In contrast, the deviations are much larger with the mutants (Supplementary Fig. S3b & c), particularly for the C-terminal region and the tetramerization loop.

**Figure 5:**
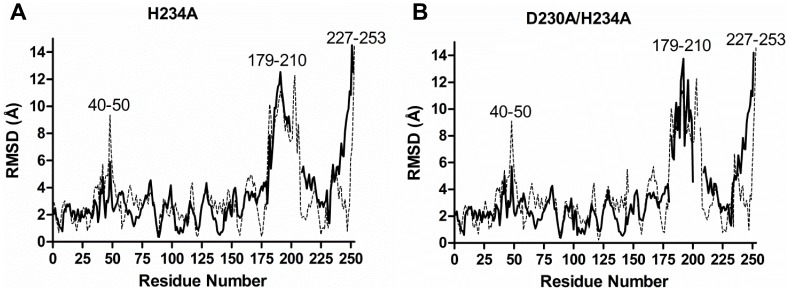
Deviations of equivalent Cα atoms after structural superposition plotted against residue number. A) Comparison of wild type and H234A mutant. B) Comparison of wild type and D230A/H234A. Results of comparing A and B chains are shown in darker and lighter shades, respectively.

The polypeptide folds of the mutants were compared to that of the native protomer by plotting deviations of corresponding Cα atoms after structural superposition (Fig. 5). The segments with large deviations between the wild type and mutant structures correspond to the swapped helix (residues 227–253), the tetramerization loop (residues and 179–210) and a loop near the active site (residues 39–51). Residues 39–51 have similar conformation in the two mutants.

### Dimeric Organization

**Figure 6:**
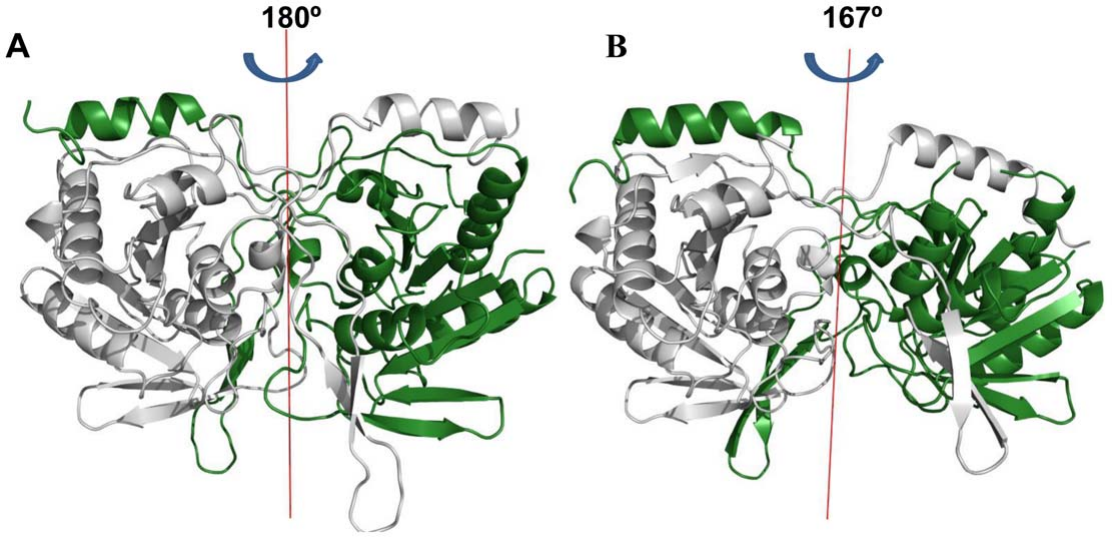
Dimeric structures of wild type and H234A mutant. A) *St*SurE. B) H234A mutant. The displacement of the rotation axis relating the two subunits in the mutant may be observed.

The two protomers in the asymmetric unit of the mutant SurEs form very similar but highly distorted dimers (Fig. 6). In both mutants, the two protomers of the asymmetric unit are related by a rotation of 167°. In order to compare dimeric organization, native and mutant dimers were superposed using the Cα positions of the A subunits. After the superposition, the direction cosines of the axes that relate A and B subunits of the native and mutant dimers were determined. The angle between these axes was ∼13.5°. Therefore, the dimers of mutant SurEs could be visualized as departures from the dimeric form of the native structure.

**Figure 7:**
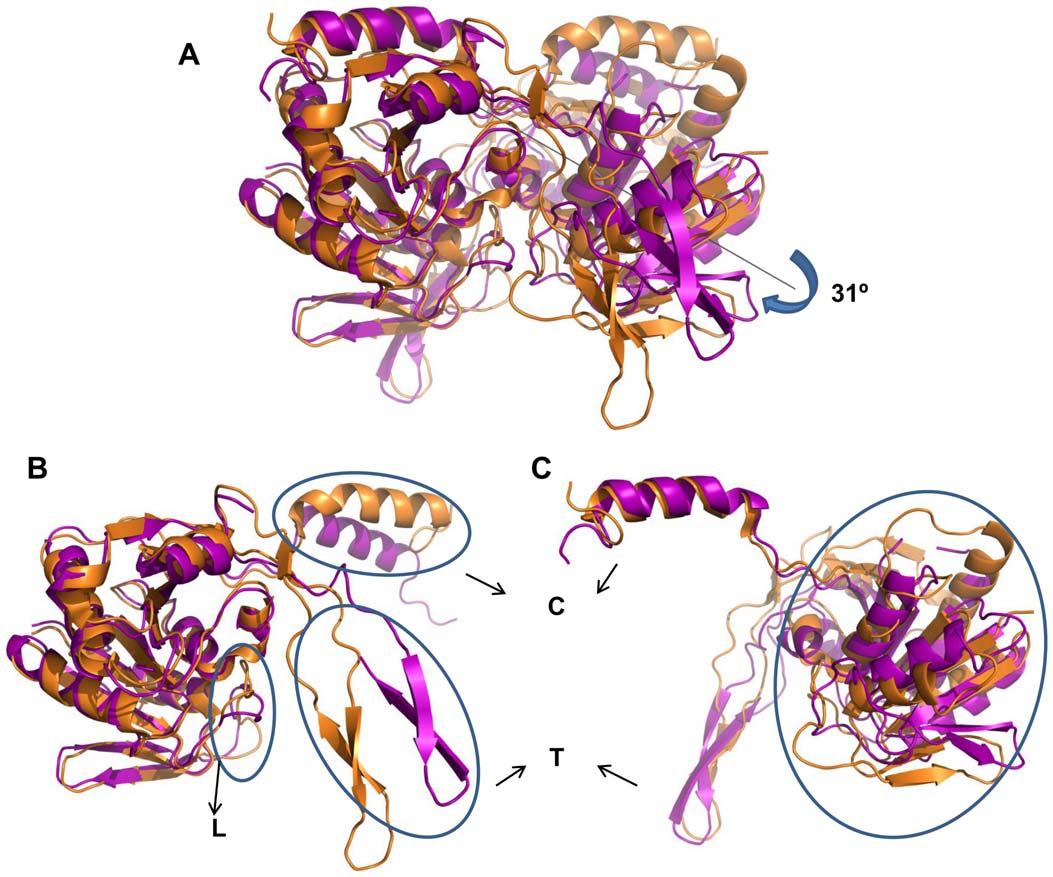
Comparison of the dimeric structure in wild type and mutant SurEs after structural alignment of A chains. A) Superposition of wild type (orange) and H234A (magenta) dimers using only A chain for structural alignment leaves 31° difference in the orientation of B-subunits. A) is shown in two parts corresponding to A and B chains respectively in B) and C). The encircled regions denote the part of the protomer where the mutant structure has departed from the wild type structure. C, T and L denotes the C-terminal helices (residues 227–253), tetramerization loop (residues 179–210) and loop near active site (residues 39–51) respectively.

When only the A chain is used for superposition (Fig. 7), the B chains of the mutant and native structures are related by a residual rotation of ∼31° (Fig. 7a and 7c). Despite this large change, both the mutants retain dimeric state in solution (Fig. 2). The dimeric interfaces in the native and mutant proteins bury comparable solvent accessible areas (Table 2). Therefore, it is not surprising that the T_m_ (Fig. 3) of the mutants is similar to that of the wild type. A segment near the active site (residues 40–45) and residues 103, 112 and 114 contribute to the dimeric interactions in the native enzyme. These interactions are absent in the mutants.

**Table 2:** Dimer Interface.

Protomers	Interface area (Å^2^)	No. of hydrogen bonds	No. of salt bridges
**Wild type (A/B dimer)**	3662	37	7
**Wild type (C/D dimer)**	3648	44	5
**H234A (A/B dimer)**	3361	32	9
**D230A/H234A (A/B dimer)**	3281	27	7

### Structural Differences between the Native and Mutant Polypeptides

In the native structure, both the tetramerization loop and the swapped C-terminal helix move away from the core of the protomer to which they belong and interact with residues of the other protomer (Fig. 1a). In the mutants, the tetramerization loop and C-terminal helix of A subunit show a relative rotation of around 31° with respect to the rest of the polypeptide (Fig. 7b) as though these segments of A subunit are intimately associated with the B subunit and hence undergo the same rotation as the B subunit. As a consequence, the interactions of the C-terminal helix and tetramerization loop with the adjacent subunit are largely retained in the mutants. Residues 200–208 are ordered in all subunits of the native enzyme. This segment is ordered only in the B subunit of both the mutants. In the A subunits of the mutants, this segment is fully (H234A) or partially (D230A/H234A) disordered. If this segment is modelled in the A subunit based on the conformation observed in the B subunit, it will have steric clashes with the tetramerization loop of a neighbouring protomer. The disorder of this segment in the mutants might therefore be due to crystal packing. In D230A/H234A, the segment 147–151 of chain B is disordered probably due to its proximity to the C-terminal helix. Apart from these segments, most other regions also show considerable changes with deviations of 2 to 4 Å between equivalent Cα atoms. Thus the mutations have not only caused large local changes but also affected the overall structure of the protomers.

D230 side chain forms intra chain hydrogen bonds with side chains of H234 and T232 in the wild type *St*SurE (Supplementary Fig. S1b). D230 OD2– H234 NE2 hydrogen bond is lost in the mutants. In chain A of H234A, D230 carboxylate is hydrogen bonded to H228 ND1 (2.90 Å). In chain B, D230 carboxylate is hydrogen bonded to T232 OG1 (2.76 Å). In addition, D230 carboxylate is also hydrogen bonded to T232 N (3.19 Å). In D230A/H234A, the loss of interactions due to mutation of Asp to Ala is compensated by the formation of an inter-chain hydrogen bond between H228 NE2/A and T232 OG1/B (2.79 Å). The distance between α carbon atoms of D230 of A and B chains in the wild type structure is around 9.5 Å. These residues are connected by hydrogen bonds involving three water molecules. In the mutant dimers, the hinge regions of the two chains are closer (Cαs of D230s are at ∼ 5 Å). This leads to the formation of several new hydrogen bonds involving D230 in H234A (D230 O/A - D230 N/B (2.85 Å) and D230 N/A – D230 O/B (3.04 Å)).

### Active Site Geometry

**Figure 8:**
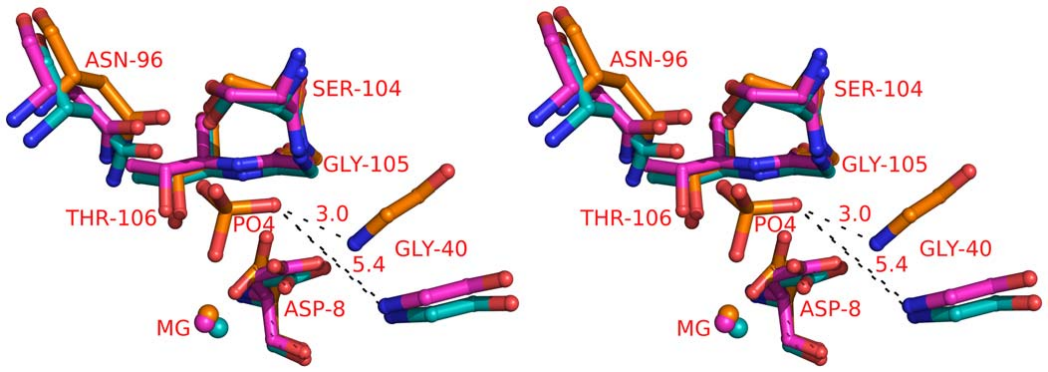
Stereo view of the active site residues of SurEs. Colour code: *St*SurE – orange, H234A – blue, D230A/H234A – magenta.

The active site of SurE has been identified based on the positions of bound magnesium and phosphate ions. The active site is close to the dimeric interface, although the catalytically important residues are from the same subunit [10]. Two negatively charged residues, D8 and D9 and a loop near active site (residues 39–51) are thought to be important for the function of *St*SurE. In the mutants, there was no density for the phosphate. Sites corresponding to Mg^2+^ ions had significant density suggesting that the cation binding site is retained in the mutants also. In the mutants, Gly40 of the loop near the active site (Fig. 8 and Fig. 7b) is displaced by >5 Å and D8 side chain is flipped away from the site of Mg^2+^ binding. Due to these changes, the active site geometry is more open when compared to that of the native SurE and does not appear to be appropriate for the phosphate binding.

## Discussion

In *St*SurE, hydrogen bonding between OD2 of D230 and NE2 of H234 imparts rigidity to the hinge region connecting the swapped helices and the rest of the monomer. The rigidity prevents the swapped helices from turning back and becoming a part of the same monomer. However, in the mutants, with the loss of hydrogen bonding, the rigidity is lost leading to a large displacement of the helical segments and loss of a large number of interactions involving the C-terminal helix. The interactions of C-terminal helices are restored by change in the relative orientations of the two protomers of the dimer, which are related by a rotation of 167° in the structures of mutants. The large rotational displacement of protomers causes similar large scale change in the orientation of the tetramerization loop involved in inter-subunit interactions. The loss of D230/OD2 - H234/NE2 hydrogen bond also leads to a large shift in the position of a loop near the active site pocket such that the mutants can no longer bind phosphate required for activity.

In the mutants, symmetry is not preserved despite the presence of an altered domain swapping suggesting that precise helix swapping interactions are necessary for the exact two fold symmetry of *St*SurE. Surprisingly, loss of symmetry and large scale structural alterations do not affect the oligomeric state of the mutants. Both *St*SurE and the mutants are dimers in solution also. In native *St*SurE, the dimer is stabilised by interactions involving the swapped helix (residues 227–253), tetramerization loop (residues 179–210) and a loop near active site (residues 39–51). In the mutants, most of these interactions are preserved as a result of concerted movements of these segments. Inter-subunit interactions involving the tetramerization loops appear to be essential for the dimeric structure in the absence of two fold symmetry.

The metal ion binding site in *St*SurE is at distances of 23 Å and 30 Å, respectively, from the mutated residues D230 and H234. Despite this large distance, the mutants were inactive. This long range effect is caused by the relay of interactions between the site of mutation and the active site. The concerted movement of swapped helix and tetramerization loop leads to displacement of the loop near the active site (residues 39–51). The 5% activity of H234A might be due to the presence in solution of a small fraction of molecules with a dimeric organization similar to that of the wild type enzyme. Thus, precise interactions between subunits brought about by domain swapping in *St*SurE is important for the symmetry, dimer organization and function of the protein.

### Conclusions

The results presented in this manuscript have revealed startling structural changes leading to loss of symmetry and function brought about by mutations that lead to loss of a crucial hydrogen bond. It would be worthwhile to examine other mutations in *St*SurE that might lead to unswapped dimeric forms or monomeric forms that are functional.

## Materials and Methods

### Cloning, Over-expression and Purification of the *St*SurE Mutants

The single site mutants D230A and H234A were constructed by site-directed mutagenesis (SDM) using recombinant *St*SurE clone as the template and expressed in invitrogen pRSETC vector. The primers for the PCR amplification of single mutants D230A and H234A were designed such that they have *HpaI* and *NheI* restriction sites, respectively. The sequences of the sense and antisense primers for D230A were 5′ACG CCG TTG CAT GTG G**CG** TTA ACC GCG3′ (bold letters indicate the bases substituted, underlined sequence shows the restriction site) and 5′CGC GGT TAA CGC CAC ATG CAA CGG CGT 3′. For the H234A mutant, the primers were 5′TTA ACC GCG **GCT** AGC GCG CAT GAT GTG G 3′ and 5′ C CAC ATC ATG CGC GCT AGC CGC GGT TAA 3′. The double mutant D230A/H234A was constructed using the D230A as the template and primers designed for H234A mutation. The mutants were screened by digesting with the respective restriction enzymes. The mutations were also confirmed by DNA sequencing.

The mutants were transformed into BL21 (DE3) *E.coli* cells. The transformed cells were inoculated to 30 ml LB (0.3 g casein, 0.15 g yeast extract and 0.15 g NaCl) pre-inoculum. After 4 h incubation, the pre-inoculum was added to 500 ml terrific broth (6 g trypton, 12 g yeast extract) and allowed to grow at 37°C till the OD at 600 nm reached 0.6. At this stage, protein expression was induced by the addition of 0.3 mM IPTG and the cells were further grown at 30°C for 6 hours. The cells were pelleted by centrifugation and dissolved in a buffer containing 50 mM Tris pH 8, 200 mM NaCl, 2% triton X 100 and 30% glycerol. The dissolved pellet was sonicated and centrifuged. The expressed mutants were soluble and hence were in the supernatant fraction. Ni-NTA beads were added to this fraction and kept for end to end rotation for 3 h. The protein bound beads were packed in a 30 ml column. Non specific proteins bound to the column were washed by passing 30 ml of 50 mM Tris pH 8, 200 mM NaCl and 20 mM imidazole. The mutant proteins were eluted with 5 ml of 50 mM Tris pH 8, 200 mM NaCl, 200 mM imidazole. Finally to remove excess imidazole, the eluted protein was dialyzed against 1 l of 50 mM Tris pH 8 and 100 mM NaCl. The yields of D230A, H234A and D230A/H234A were 0.6 mg, 3 mg and 1.3 mg, respectively, per litre of culture.

### Crystallization, Data Collection and Processing

H234A and the double mutant D230A/H234A of *St*SurE were crystallised by under oil microbatch method using 1∶1 mixture of silicon and paraffin oils to cover the crystallization droplets. H234A at a concentration of 4.5 mg/ml in 0.1 M HEPES pH 7.5, 0.02 M MgCl_2_ hexahydrate was crystallized by precipitation with 22% (w/v) polyacrylic acid 5100 sodium salt. D230A/H234A at a concentration of 1.2 mg/ml in 0.1 M HEPES pH 7.5, 0.02 M MgCl_2_ hexahydrate, 30% (w/v) D-glucose was similarly crystallized by the addition of 22% (w/v) polyacrylic acid 5100 sodium salt. A data set extending to 2.1 Å resolutions was collected at 100K on a H234A mutant crystal using the BM14 beam line of ESRF synchrotron, Grenoble, France. A 2.35 Å resolution dataset was collected at 100K on a crystal of D230A/H234A double mutant using a Rigaku RU300 rotating anode X ray generator and a MAR research imaging plate detector. Both the data sets were processed using the MOSFLM [19] and scaled by SCALA [20] programs of CCP4 suite [16]. The data processing statistics are shown in Table 1.

### Structure Determination, Validation and Analysis

The structures of mutants were determined by Molecular Replacement (MR) using the Phaser program [14]. The structures were refined with REFMAC program [15] of CCP4 suite [16]. The resulting structures were examined and manually built using COOT [17]. Water molecules were identified using REFMAC and manual examination of difference electron density maps. Final models obtained after alternative cycles of model building and refinements were validated using PROCHECK [18]. Average B factors were calculated using BAVERAGE program of CCP4 suite [16]. Structural alignments were achieved using ALIGN [21], Superpose [16] and SSM superpose option of COOT [22]. Illustrations of structures were produced using PyMOL [23]. Multiple sequence alignment of SurE homologs was achieved using ClustalW [24]. A graphical representation of the alignment was obtained using ESPript [25]. The Protein Interfaces, Surfaces and Assemblies (PISA [26]) server was used for the analysis of dimeric interfaces.

### Alkaline Phosphatase Activity

The alkaline phosphatase activity of the native and mutant enzymes was estimated using *p*-Nitrophenyl phosphate (pNPP) as a synthetic substrate. The protein was first dialyzed against a buffer containing 50 mM Tris pH 8, 100 mM NaCl, 10 mM EDTA. The reaction mixture of 150 µl contained 15 mM pNPP, 100 mM cacodylate pH 7, 10 mM MgCl_2_ and 100 ng of *St*SurE or 500 ng H234A or 1500 ng of D230A/H234A. The concentration of protein was estimated by measurement of OD at 280 nm. The native enzyme served as the positive control. After incubation of the mixture at 37°C for 30 min, the reaction was quenched by adding 100 µl 2 M NaOH. The yellow coloured *p-*Nitrophenol product formed during the reaction was estimated by recording the OD at 405 nm. The OD was corrected for absorption by residual substrate. A standard curve of *p*-Nitrophenol (micromoles) versus the OD at 405 nm was plotted. From this plot, product formed in micromoles corresponding to the measured OD was determined. The enzyme activity was expressed as µmoles of product formed per min of the reaction per µg of enzyme.

### Biophysical Characterization

Circular dichroism (CD) spectra of *St*SurE and mutants were recorded using a Jasco J715 spectropolarimeter. Protein at a concentration of 0.3 mg/ml in 5 mM HEPES pH 7.5 was used in a cell with a path length of 1 mm to record the data at every 0.5 nm with a response time of 4 s and a bandwidth of 2 nm. Thermal denaturation was monitored by recording the CD band at 208 nm from 20 to 100°C with a rate of 1°C/min rise in temperature in a Peltier cell holder (Jasco). The oligomeric state of the proteins (1.7 mg/ml, 200 µl) was examined by gel-filtration chromatography on a S200 analytical column with a bed volume of 24 ml. Dynamic light scattering (DLS) measurements were performed on a DynaPro DLS instrument using a 45 µl cuvette with a data acquisition time of 0.7 s for each of the 100 readings. DynamicsV6 software was used to calculate the radius of gyration R_H_.

### Accession Numbers

Coordinates and structure factors of H234A and D230A/H234A are deposited in the Protein Data Bank with codes 4G9O and 4GAD respectively.

## Supporting Information

Figure S1 **Identification of residues promoting C-terminal helix swapping in** ***St*** **SurE.** Comparison of sequences of C-terminal variable domain of SurE’s. The secondary structures of domain swapped segments are highlighted. Sequences were aligned using ClustalW and representation of the aligned sequences and secondary structures was obtained by ESPRIPT. 2V4N, 2WQK, 1J9J, 2E6E, 3TY2 and 1L5X are PDB codes of *St*SurE, *Aa*SurE, *Tm*SurE, *Tt*SurE, *Cb*SurE and *Pa*SurE, respectively. Arrows indicate the residues that were mutated. B) and C) illustrate interactions stabilising the hinge and favouring swapping of C-terminal helices in *St*SurE and *Aa*SurE, respectively.
(TIF)

Figure S2 **Fit of mutated residues.** A) 2Fo-Fc (1σ) map corresponding to residues 230 and 234 in native *St*SurE. B) 2Fo-Fc (1σ) map corresponding to residue 234 from chains A (gray) and B (green) of H234A mutant. C) and D) shows the 2Fo-Fc (0.8σ) map of residues 230 and 234 respectively, in D230A/H234A.
(TIF)

Figure S3 **Deviations of equivalent Cα atoms of A and B chains after structural superposition plotted against residue number.** A) Wild type *St*SurE. B) H234A. C) D230A/H234A.
(TIF)
