# Supplementary figures and images for: Dramatic Structural Changes Resulting from the Loss of a Crucial Hydrogen Bond in the Hinge Region Involved in C-Terminal Helix Swapping in SurE: A Survival Protein from Salmonella typhimurium
Source: PLoS One. 2013 Feb 7;8(2):e55978. doi: 10.1371/journal.pone.0055978 (PMC3567009; doi:10.1371/journal.pone.0055978)

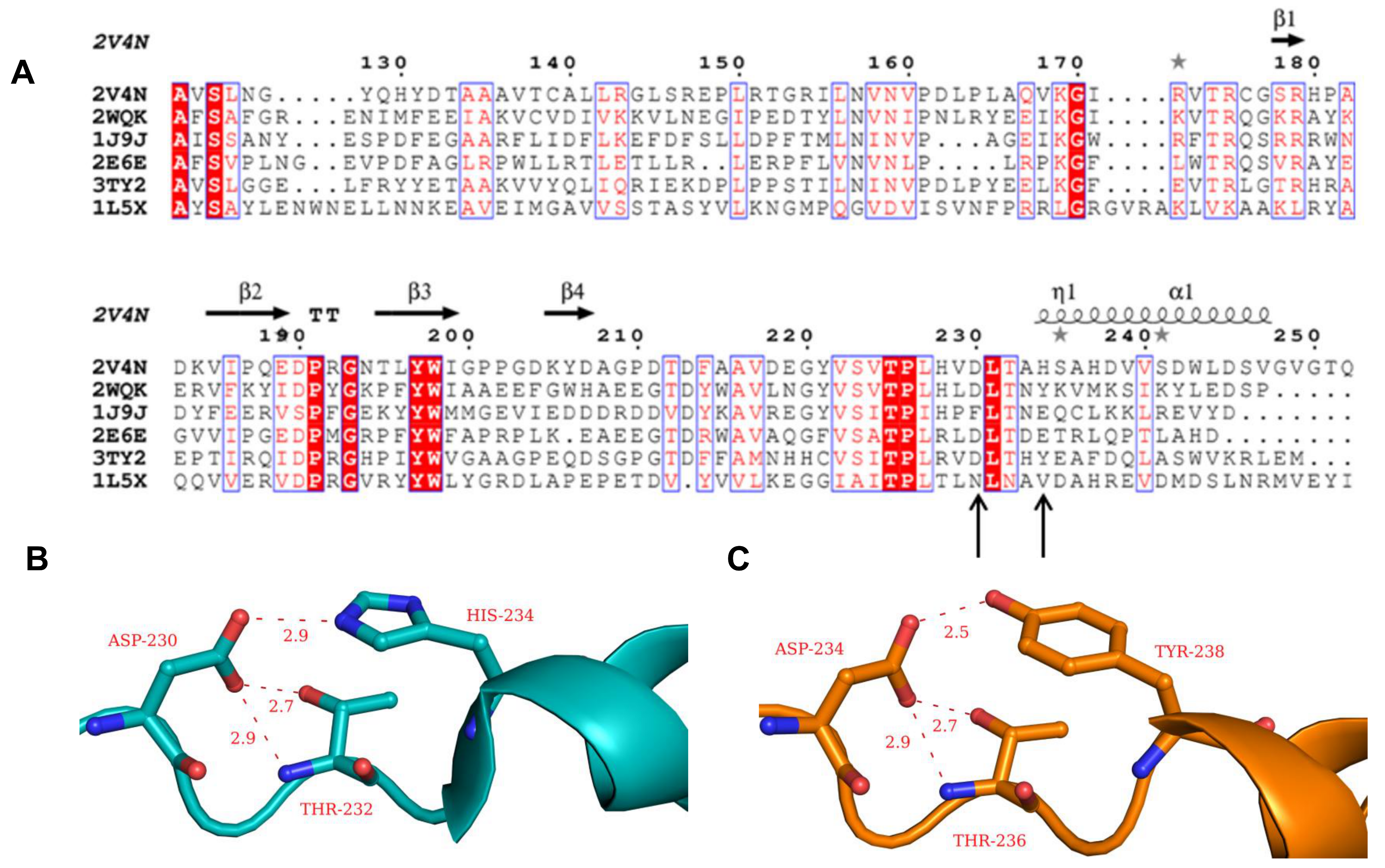

Supplement: Figure S1 — Identification of residues promoting C-terminal helix swapping in St SurE. Comparison of sequences of C-terminal variable domain of SurE’s. The secondary structures of domain swapped segments are highlighted. Sequences were aligned using ClustalW and representation of the aligned sequences and secondary structures was obtained by ESPRIPT. 2V4N, 2WQK, 1J9J, 2E6E, 3TY2 and 1L5X are PDB codes of StSurE, AaSurE, TmSurE, TtSurE, CbSurE and PaSurE, respectively. Arrows indicate the residues that were mutated. B) and C) illustrate interactions stabilising the hinge and favouring swapping of C-terminal helices in StSurE and AaSurE, respectively. (TIF) [file pone.0055978.s001.tif]

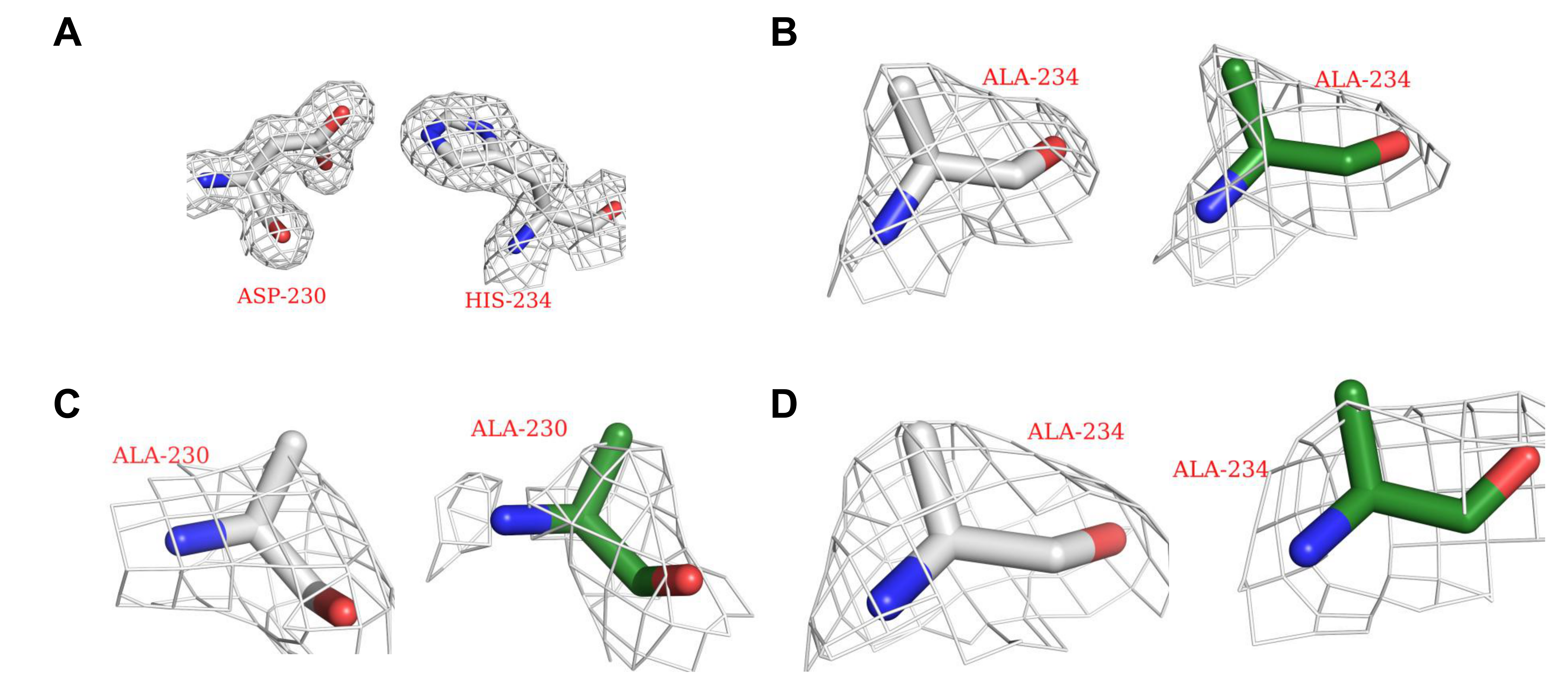

Supplement: Figure S2 — Fit of mutated residues. A) 2Fo-Fc (1σ) map corresponding to residues 230 and 234 in native StSurE. B) 2Fo-Fc (1σ) map corresponding to residue 234 from chains A (gray) and B (green) of H234A mutant. C) and D) shows the 2Fo-Fc (0.8σ) map of residues 230 and 234 respectively, in D230A/H234A. (TIF) [file pone.0055978.s002.tif]

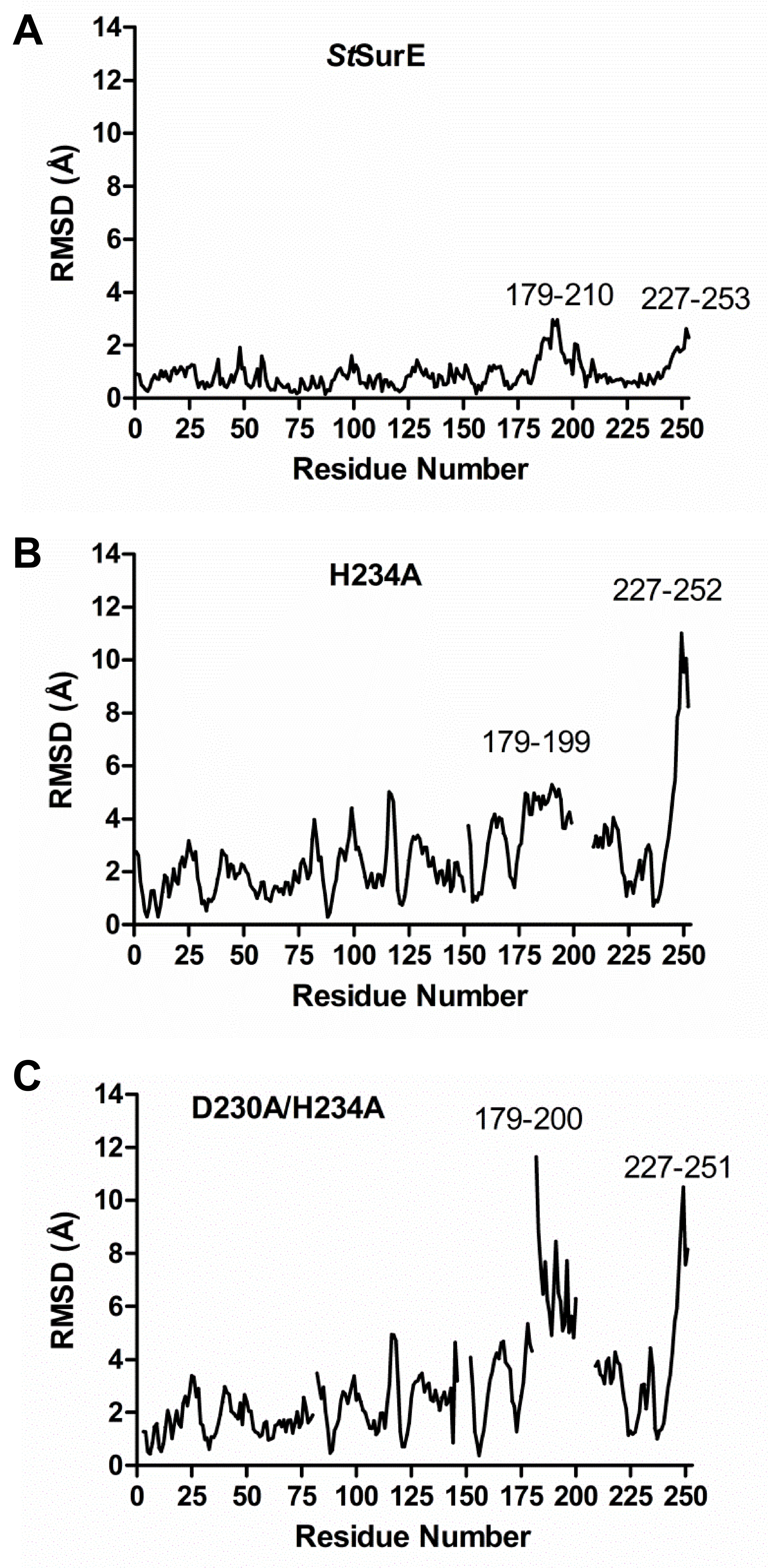

Supplement: Figure S3 — Deviations of equivalent Cα atoms of A and B chains after structural superposition plotted against residue number. A) Wild type StSurE. B) H234A. C) D230A/H234A. (TIF) [file pone.0055978.s003.tif]
